# Fear of COVID-19 disease and vaccination as predictors of vaccination status

**DOI:** 10.1038/s41598-023-35064-0

**Published:** 2023-05-31

**Authors:** Donya Gilan, Markus Birkenbach, Marius Wossidlo, Philipp Sprengholz, Cornelia Betsch, Omar Hahad, Klaus Lieb

**Affiliations:** 1 Leibniz Institute for Resilience Research Mainz, Mainz, Germany; 2 Department of Psychiatry and Psychotherapy, University Medical Center Mainz, Mainz, Germany; 3 Department of Psychology, Johannes Gutenberg University Mainz, Mainz, Germany; 4 Institute for Planetary Health Behavior, University of Erfurt, Erfurt, Germany; 5 Bernhard Nocht Institute for Tropical Medicine, Hamburg, Germany; 6 Department of Cardiology, Cardiology I, University Medical Center Mainz, Mainz, Germany; 7 German Center for Cardiovascular Research (DZHK), Partner Site Rhine-Main, Mainz, Germany

**Keywords:** Public health, Human behaviour

## Abstract

Vaccination rates are still insufficient to prevent the spread of COVID-19, so immunity must be increased among the population in order to reduce the virus’ spread and the associated medical and psychosocial effects. Although previous work has identified various factors associated with a low willingness to get vaccinated, the role of emotions such as fear of vaccination (FVAC) or fear of COVID-19 (FCOV), vaccination as a subjective norm (SN), psychological factors like general control beliefs (CB) or psychological resilience, and their interaction have been investigated less intensively. We used data from three cross-sectional waves of the German Panel COSMO (November 2021, N = 1010; February 2022, N = 1026; March 2022, N = 1031) and multiple logistic regression analyses to test whether vaccination rates are moderated by those factors. After controlling for covariates (age, sex, confidence in own intuition, optimism, well-being), we found that CB was no significant predictor of vaccination status. Higher FCOV and higher ratings in SN, however, were associated with an increased likelihood of being vaccinated. In contrast, higher FVAC was associated with a decreased likelihood of being vaccinated. Psychological resilience did not consistently moderate the associations between fear and vaccination status.

## Introduction

Rapidly, the SARS CoV-2 virus has caused a worldwide pandemic. The vaccination rate is still not sufficient in many countries, so the risk of the spread of the virus and the emergence of new viral variants remains. An acquired immunity in large parts of the population is required to reduce serious courses of infection, to minimize possible permanent adverse health consequences (e.g. long covid) and to prevent a collapse of the health care system^1^.

In order to increase the willingness to vaccinate, various strategies have been developed, e.g., education through vaccination campaigns, easy access to vaccination in vaccination centers, role models (e.g., celebrities) who promote vaccination, and low-threshold vaccination services (e.g. mobile vaccination teams). The 5C model^2,3^ poses five key aspects that can influence a person's vaccination behavior: (1) *Confidence* (in efficacy, safety and the health care system), (2) *Constraints* (availability, accessibility), (3) *Complacency* (personal perception of own infection risks), (4) *Calculation* (personal risk–benefit assessment), and (5) *Collective Responsibility* (willingness to participate in collective efforts)^2,3^. Indeed, according to an early experimental study, lifting restrictions for vaccinated persons, financial incentives, and vaccination by general practitioners led to an overall increase in the willingness to vaccinate^4^. In particular, according to a pre-registered randomized clinical trial, financial incentives increased vaccine uptake by about 4 percentage points^5^ and have no unintended negative consequences^6^. Also, students who are active in informal relationships, work in the mental field, used psychological/psychiatric services before the pandemic and who study medicine showed a higher willingness to be vaccinated^7^. There are cultural differences in the reasons for unwillingness to vaccinate, but some universal reasons (e.g. “I am concerned about the serious side effects of the vaccine”) can be identified^7^. Due to the perception of the pandemic as severe, confidence in the safety of the vaccine, lower financial concerns, lower stigma, higher pro-social attitudes, and confidence in health authorities, more than 95% of employees were willing to be vaccinated^8^. Insufficient information and skepticism about the vaccines were the main reasons for unwillingness to pay, although long-term side effects, duration of immunity and mortality rate were important decision attributes^9^. However, pregnant women and mentally ill people are often willing to pay or pay more for a vaccine^10,11^.

Moreover, reminder texts increased vaccination rates by an average of 2 percentage points or 6.8% over a 3-month follow-up period^12^. Other study results highlight the positive effect of providing information about the safety and efficacy of the new COVID-19 vaccines on vaccination intent^13^. Also, a previous COVID-19 disease led to higher vaccination intention via the mediation of fear^14^. Living with people in poor health, viewing vaccinations as subjective or moral norms, and perceiving vaccinations as beneficial and necessary were also associated with higher willingness to vaccinate among the unvaccinated^15^. Positive attitudes towards vaccines and previous vaccination were positively related to vaccination intention, whereas safety concerns negatively influenced vaccination intention^14^. There are additional influential psychological factors that have not been widely considered in the context of vaccine mobilization.

**Figure 1:**
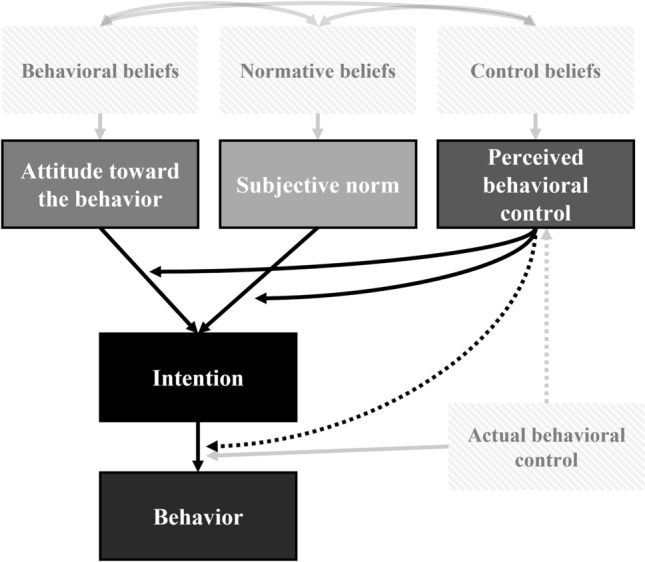
The theory of planned behavior^16^. *Note*: Figure based on the graphic from Ajzen^18^. Behavioral, normative and control beliefs as well actual behavioral control (cross-hatched boxes) only serve to complete the theory and will not be considered further, in contrast to the black and grey boxes (e.g. Intention).

If the purpose is to enhance people's motivation to behave healthily, the theory of planned behavior^16^ comes into focus as an explanation framework. The theory states that the intention to perform a certain behavior (and subsequently the behavior itself) can be predicted by three factors: *behavioral beliefs, normative beliefs,* and *control beliefs* (see Fig. 1). Behavioral beliefs can lead to a positive or negative attitude toward the behavior, whereas normative beliefs can lead to perceived pressure or to a subjective norm and behavioral control or self-efficacy are in turn outcomes of control beliefs^16,17^.

Affective determinants such as fear, part of the attitude component of the theory of planned behavior, have a relevant impact on the willingness to vaccinate. A longitudinal study by Mertens, et al.^19^ investigated whether fear of COVID-19 predicts willingness to vaccinate. They showed that increased fear of COVID-19 predicted willingness to be vaccinated 14 months later, even when several anxious personality traits, perceptions of infection control, risks to relatives, self-rated health, previous infections, media use, and demographic variables were controlled for. Another study examined the extent to which existential fears (subjective closeness to death and fear of death) were related to COVID-19 vaccination fear and demonstrated that both existential fears were positively associated with COVID-19 vaccination fear, and that the level of fear of death moderated the positive association between subjective closeness to death and COVID-19 vaccination fear^20^.

Given that fear of vaccinations appears to be an important predictor of vaccinations, emotion-regulated coping mechanisms affecting vaccination readiness might be an interesting area of research. At present, however, only few studies have been conducted on functional coping mechanisms and fear of vaccinations. One promising study reported that self-efficacy was the strongest predictor that was associated with lower vaccine hesitancy^21^.

In this study, we used the theory of planned behavior as a framework to identify important predictors of COVID-19 vaccination status. We focused on how fear of COVID-19 as well as fear of COVID-19 vaccination (attitudes), subjective norms, and control belief (perceived behavioral control) are associated with vaccination status. We also built on preliminary work investigating the role of coping mechanisms as a moderator between fear and vaccination status^20,21^. We hypothesize that (1) the more vaccinations are viewed as social norms, the more a person is convinced that they control events in their life, the more a person fears COVID-19, and the less a person fears vaccinations, the more likely it is that a person is vaccinated. Additionally, we expect that (2) fear of COVID-19 and vaccination status is moderated by resilience in the direction that people with high fear of COVID-19 vaccination, but high psychological resilience are more likely to be vaccinated and vice versa, people with high fear of COVID-19 but high psychological resilience are less likely to be vaccinated.

## Results

### Sample

In the first survey (November 30, 2021), five participants were excluded from analysis since they terminated questionnaire completion early which left us with *N* = 1010 participants. The sample included 502 men (49.7%) and 508 women (50.3%). 459 participants (45.4%) finished lower secondary education, 551 (54.6%) finished at least upper secondary education, and 891 participants (88.2%) were vaccinated. Participants were between 18 and 74 years old, with a mean age of *M* = 44.86 years (*SD* = 15.69). In the second survey (February 22, 2022) we received data from *N* = 1026 participants. The sample included 519 men (50.6%) and 507 women (49.4%). 462 participants (45.0%) finished lower secondary education, 564 (49.4%) finished at least upper secondary education, and 920 participants (89.7%) were vaccinated. Participants were between 18 and 74 years old with a mean age of *M* = 45.3 years (*SD* = 16.11). In the third survey (March 15, 2022) we received data from *N* = 1031 participants. The sample included 506 men (49.1%) and 525 women (50.9%). 433 (41.0%) finished lower secondary education, 608 (59.0%) finished at least upper secondary education, and 930 participants (90.2%) were vaccinated. Participants were between 18 and 74 years old, with a mean age of *M* = 45.0 years (*SD* = 15.6). There were no significant differences between samples in age (*F*(2, 2042.1) = 0.19, *p* = 0.825), gender (χ^2^(2) = 2.41, *p* = 0.300), education (χ^2^(2) = 3.74, *p* = 0.154), or proportion of vaccinated participants (χ^2^(2) = 2.26, *p* = 0.323).

Compared to vaccinated participants, unvaccinated participants saw vaccination as less of a social norm (Cohen’s *d* between 1.20 and 1.44), reported less fear of COVID-19 (Cohen’s *d* between 0.67 and 0.90), and reported higher fear of a COVID-19 vaccination (Cohen’s *d* between 1.70 and 2.12), while there was no difference in general control belief (Table 1).

**Table 1:** Comparisons between vaccinated and unvaccinated participants.

	Vaccinated		Unvaccinated		*t*	*p*	*d*
	*M*	*SD*	*M*	*SD*			
Sample 1							
N	891		119				
Fear of COVID-19	4.64	1.68	3.08	2.02	8.01	< 0.001	0.90
Fear of COVID-19 vaccination	1.90	1.57	4.76	2.34	− 12.91	< 0.001	− 1.70
Vaccination as social norm	5.48	1.54	3.57	1.92	10.40	< 0.001	1.20
Control belief	2.49	1.06	2.57	1.12	− 0.75	0.453	− 0.08
Sample 2							
N	920		106				
Fear of COVID-19	3.70	1.74	2.54	1.70	6.52	< 0.001	0.67
Fear of COVID-19 vaccination	1.96	1.64	5.30	2.07	− 16.06	< 0.001	− 1.98
Vaccination as social norm	5.32	1.63	3.33	1.86	10.60	< 0.001	1.21
Control belief	2.39	1.06	2.40	1.13	− 0.08	0.940	− 0.01
Sample 3							
N	930		101				
Fear of COVID-19	3.69	1.78	2.26	1.64	7.75	< 0.001	0.81
Fear of COVID-19 vaccination	1.84	1.54	5.25	2.08	− 16.00	< 0.001	− 2.12
Vaccination as social norm	5.51	1.58	3.20	1.84	12.15	< 0.001	1.44
Control belief	2.35	1.05	2.46	1.14	− 0.92	0.359	− 0.10

*d* = Cohen’s d effect size. Means of all reported variables may vary between 1 and 7.

### Regression analyses

Correlation analyses revealed no multicollinearity. With a Nagelkerke R^2^ between 0.430 and 0.517, our model had medium to large overall effect sizes in all samples^22^. In all three samples, vaccination status was consistently predicted by vaccination as social norm, fear of COVID-19, and fear of COVID-19 vaccination (Table 2). General control belief did not significantly predict vaccination status. Participants with higher ratings of fear of COVID-19 (OR between 0.729 and 0.794) as well as viewing vaccinations as a subjective norm (OR between 0.694 and 0.717) were more likely to be vaccinated, while participants with higher ratings of fear of a COVID-19 vaccination (OR between 1.701 and 1.910) were more likely to be unvaccinated. This means that, on average, an increase in viewing vaccination as a subjective norm by 2.0 scale points or an increase in fear of COVID-19 by 2.4 scale points doubles the probability of being vaccinated while an increase in fear of a COVID-19 vaccination by 1.2 scale points doubles the probability of being unvaccinated.

**Table 2:** Logistic regression analysis predicting vaccination status.

Variable	*B*	*SE*	Wald	*p*	*OR*	95% CI	
						Lower bound	Upper bound
Sample 1							
Fear of COVID-19	− 0.31	0.07	21.71	< 0.001	0.734	0.644	0.836
Fear of COVID-19 vaccination	0.53	0.06	87.78	< 0.001	1.701	1.522	1.901
Vaccination as social norm	− 0.34	0.07	25.15	< 0.001	0.711	0.622	0.812
Control belief	− 0.18	0.11	2.55	0.111	0.836	0.670	1.042
Nagelkerke R^2^	0.430						
Sample 2							
Fear of COVID-19	− 0.23	0.08	8.83	0.003	0.794	0.682	0.925
Fear of COVID-19 vaccination	0.65	0.06	107.96	< 0.001	1.910	1.691	2.158
Vaccination as social norm	− 0.33	0.07	20.49	< 0.001	0.717	0.620	0.828
Control belief	− 0.12	0.12	1.09	0.296	0.883	0.700	1.115
Nagelkerke R^2^	0.470						
Sample 3							
Fear of COVID-19	− 0.32	0.08	14.14	< 0.001	0.729	0.618	0.859
Fear of COVID-19 vaccination	0.63	0.06	97.93	< 0.001	1.881	1.660	2.132
Vaccination as social norm	− 0.37	0.07	24.36	< 0.001	0.694	0.600	0.802
Control belief	0.07	0.13	0.28	0.596	1.070	0.833	1.376
Nagelkerke R^2^	0.506						

*B* regression weights, *SE* standard error of regression weights, *OR* odds ratios.

Control analyses that included wellbeing, need for cognition, trust in own intuition, optimism, age, and gender as covariates confirmed the robustness of these results. None of the added covariates consistently predicted vaccination status while fear of COVID-19, vaccination as socially wanted norm, and fear of a COVID-19 vaccination remained significant predictors with virtually identical odds ratios as without the included covariates. For detailed results see Supplementary Table 1.

In our next analysis step, we tested whether the associations between fear of COVID-19 and vaccination status as well as fear of a COVID-19 vaccination and vaccination status are moderated by self-reported psychological resilience. In a not preregistered preliminary analysis we calculated the association between psychological resilience and vaccination status and found a weak point-biserial correlation (r_pb_ = − 0.67, *p* = 0.032) in the first sample, and no significant association in the other samples. The final moderated logistic regression model again had a good to very good fit with a Nagelkerke R^2^ between 0.445 and 0.520. Our results showed that neither the association between fear of COVID-19 and vaccination status nor the association between fear of COVID-19 vaccination and vaccination status were moderated by psychological resilience. The only exception was the first sample where resilience did moderate the association between fear of COVID-19 and vaccination status. However, this moderation was not replicated in the two other samples (Table 3).

**Table 3:** Testing interaction Fear of COVID-19 × Resilience and Fear of COVID-19 vaccination x Resilience in predicting vaccination status.

Variable	*B*	*SE*	Wald	*p*	*OR*	95% CI	
						Lower bound	Upper bound
Sample 1							
Fear of COVID-19	− 0.33	0.07	23.19	< 0.001	0.717	0.626	0.821
Fear of COVID-19 vaccination	0.55	0.06	88.53	< 0.001	1.741	1.551	1.954
Vaccination as social norm	− 0.34	0.07	24.60	< 0.001	0.713	0.624	0.815
Control belief	− 0.20	0.11	3.05	0.081	0.821	0.658	1.024
Resilience	− 0.08	0.21	0.15	0.701	0.923	0.615	1.386
Fear of COVID-19 × Resilience	− 0.26	0.08	9.52	0.002	0.771	0.653	0.909
Fear of a COVID-19 vacciation × Resilience	− 0.03	0.07	0.24	0.627	0.967	0.845	1.106
Nagelkerke R^2^	0.445						
Sample 2							
Fear of COVID-19	− 0.21	0.08	7.31	0.007	0.808	0.692	0.943
Fear of COVID-19 vaccination	0.66	0.06	103.34	< 0.001	1.931	1.701	2.192
Vaccination as social norm	− 0.35	0.08	21.19	< 0.001	0.706	0.609	0.819
Control belief	− 0.11	0.13	0.70	0.403	0.900	0.704	1.152
Resilience	0.39	0.26	2.28	0.131	1.483	0.889	2.474
Fear of COVID-19 × Resilience	0.10	0.09	1.13	0.287	1.100	0.923	1.312
Fear of a COVID-19 vaccination × Resilience	− 0.09	0.07	1.64	0.200	0.912	0.792	1.050
Nagelkerke R^2^	0.475						
Sample 3							
Fear of COVID-19	− 0.31	0.09	13.17	< 0.001	0.734	0.621	0.867
Fear of COVID-19 vaccination	0.64	0.07	96.13	< 0.001	1.897	1.669	2.157
Vaccination as social norm	− 0.35	0.07	22.50	< 0.001	0.701	0.606	0.812
Control belief	0.10	0.13	0.60	0.439	1.110	0.853	1.445
Resilience	0.28	0.28	0.95	0.330	1.319	0.756	2.303
Fear of COVID-19 × Resilience	0.10	0.10	0.89	0.346	1.104	0.899	1.355
Fear of a COVID-19 vacciation × Resilience	0.00	0.08	0.00	0.974	0.997	0.852	1.168
Nagelkerke R^2^	0.520						

B = regression weights, SE = standard error of regression weights, OR = odds ratios.

Control analyses including wellbeing, need for cognition, trust in own intuition, optimism, age, and gender as covariates showed the robustness of these results since the pattern of results did not change (i.e., the moderation remained non-significant, and odds ratios were stable). For detailed results see Supplementary Table 2.

## Discussion

The present study examined the extent to which fear of vaccination, fear of COVID-19 disease, vaccination as a subjective norm, and control beliefs (as central variables in the theory of planned behavior) predicted vaccination status. Results showed in all three samples that vaccination status was consistently predicted by vaccination as a subjective norm, fear of COVID-19, and fear of COVID-19 vaccination, whereas this was not the case for general control beliefs. It was shown that participants with higher fear of COVID-19 as well as the belief that vaccination is a subjective norm were more likely to be vaccinated. In contrast, participants with higher fear of a COVID-19 vaccination were more likely to be unvaccinated. These results are largely consistent with previous studies^19^ showing that anxiety is a relevant construct for predicting and possibly influencing vaccination readiness. Importantly, we also showed that fear of a COVID-19 vaccination is negatively associated to vaccination status, underlining that addressing fears might be useful in increasing vaccination uptake. Contrary to our hypotheses, we found no main effect of control belief, which might be due to our operationalization. We asked participants about their belief that their life is controlled by others, while their personal belief that they have freedom of choice to receive a vaccination and beliefs about the outcomes of that vaccination might have been better suited measurements.

We similarly tested the moderation effect of psychological resilience on the association of fear of COVID-19 and vaccination status and fear of COVID-19 vaccination and vaccination status. We assumed that individuals with a high fear of vaccination and high resilience would be more likely to be vaccinated due to “better” coping with this fear (or conversely, with a high fear of COVID-19, strong resilience would make vaccination less likely). We only found suggestive evidence indicating a moderating effect of resilience in the first sample, whereas results for the other samples indicated that none of the associations were moderated by resilience. The fact that we were unable to detect a moderation effect or replicate it in the subsequent waves can possibly be explained by the survey methods used. The Brief Resilience Scale (BRS)^23^ captures only one of many facets of resilience. While it is a reliable way of surveying resilience as the ability to recover from stress and can provide unique and important information about people coping with health-related stressors, it does not capture the protective factors or resources that include personal characteristics and coping styles. Therefore, the different operationalisation of resilience in our case as an outcome of coping with stress or as psychological resilience measured by the BRS may be a reason why we could not detect an effect. Other survey instruments for resilience with other underlying resilience concepts (e.g. resilience as a composition of different resilience factors) or fear-specific coping mechanisms might be better suited to test our hypothesis. Further studies are therefore needed to examine the interplay of resilience with the outcomes of interest.

Even though 76.4% of the German population has basic immunization according to official data^24^, it is fundamentally important to convince the remaining unvaccinated population to be vaccinated, especially in view of future COVID-19 waves or even new pandemics. So how can we increase the vaccination status? According to Brewer^25^, further supported by our results, studies suggest that thoughts and feelings are linked to vaccination uptake. Perceived risk of harm from infectious diseases and confidence in the safety and efficacy of vaccines are among these constructs. Additionally, interventions in general have not shown that changing thoughts and feelings increases vaccination uptake^25^. However, according to Brewer^25^ social processes such as social norms, altruism and social media sharing may be promising intervention targets in studies. Also, according to him, interventions (such as reminders, defaults, and vaccination requirements) that directly change behavior—without trying to change how people think or feel or their social experiences—are reliable effective means of increasing vaccination rates. The most effective intervention to increase vaccination rates remains the recommendation of a health care provider^25^. Our results further add to that perspective, since differences in the ability to cope with stressful life events (i.e., resilience) did not change the association between fear and the decision to get vaccinated.

Overall, our results further suggest that targeting fear and social norms in the population could play a significant role in increasing vaccination rates. While it has been shown that interventions targeting feelings toward vaccination usually show little effect, the large effects we found for the association between fear and vaccination status also show that an improved understanding of factors that drive fear is essential. Further efforts to reduce fear of (COVID-19) vaccinations have huge potential in increasing vaccination uptake and should not be dismissed because they have not been successful so far. Another important insight provided by our data might explain why interventions targeting social norms on a population level might be ineffective: A Person's view of what people close to them believe about vaccines might be more important than how vaccines are viewed in general. Interventions might therefore benefit from a more community-focused approach compared to an untargeted country-wide campaign. Additionally, resilience might be an important mechanism of action that could lead to a downregulation of fear. However, future studies need to observe the action-based aspect of resilience (i.e., coping) in this context to explore whether a moderating effect exists but wasn’t found in our data due to coarse operationalizations. Additionally, controlling for other factors potentially contributing to vaccination uptake, such as medical and psychiatric conditions, might help further clarify the role of psychological factors.

The study results add to existing evidence by being the first study to show the association between actual vaccination behavior and several psychological variables embedded in an established theoretical framework. While other studies looked at different psychological mechanisms and COVID-19 vaccination, they often did so looking at vaccination intention as an outcome (e.g.^14,19,26^), to which we add by highlighting the association between psychological mechanisms and actual behavior (vaccination). Furthermore, our study adds to the literature by considering the contradictory paths namely fear of COVID-19 as well as fear of COVID-19-vaccination and therefore allowing for a comparative evaluation of their association with vaccination rates. We further provide adjustment for several covariates as well as a moderation analysis evaluating the role of stress recovery (resilience), which has not been done prior to our knowledge. We believe that this is an important point, even though our results on stress recovery were inconclusive, but may strengthen future research efforts. Further strengths of the present study include its use of several large cross-sectional samples over an extended period during the pandemic in representative samples of the German population. However, the small number of items in the questionnaires limits our conclusions, as locus of control beliefs or resilience are multifaceted constructs and more differentiated measures might have enabled more differentiated insights.

Finally, to increase vaccination rates, programs need to be extended to address such constructs as fears associated with COVID-19 disease and vaccination and vaccination as a socially wanted norm. To do this, though, programs must be designed and piloted.

## Methods

### Participants

This investigation is part of the “COVID-19 Snapshot Monitoring” (COSMO-) study, which was initiated in Germany on March 3, 2020, to gain behavioral and cognitive insights into the German population’s reaction to the SARS-CoV-2 pandemic^27^. The quota-samples, representative for the German population regarding age and gender, were drawn from the panel supplier *respondi*’s actively managed panel. The questionnaire including was sent to around 5000 people at each of the three starting dates (November 30, 2021; February 22, 2022; March 15, 2022), to be completed within 48 h. Samples are cross-sectional and non-overlapping. The study was approved by the institutional review board at the University of Erfurt (#20200302/20200501). All participants provided informed consent before entering the study. The study was conducted according to the guidelines of the Declaration of Helsinki.

### Study variables

The theory of planned behavior states that behavioral intention and subsequently behavior, are determined by attitudes, subjective norms, and perceived behavioral control^16^. The behavior of interest, vaccination status was surveyed by asking whether participants already received a vaccine shot against COVID-19. We grouped everyone having received at least one vaccine shot in the “vaccinated” group, and everyone having received no vaccine shot in the “unvaccinated” group. We assessed Fear of COVID-19 as well as Fear of COVID-19 vaccination as measures of attitude. Fear of COVID-19 was measured with one item “To me, the Coronavirus is …” on a scale ranging from 1 “scary” to 7 “not scary”. Ratings were inverted so that higher values represent higher fear of COVID-19. Fear of a COVID-19 vaccination was measured with one item “I’m afraid of getting vaccinated against COVID-19” on a scale ranging from 1 “totally disagree” to 7 “totally agree”. Vaccination as a social norm was measured with one item “People close to me believe that you should get vaccinated” on a scale ranging from 1 “totally disagree” to 7 “totally agree”. As a global approximation of perceived behavioral control, we assessed control belief. Control belief was measured with one item from the Internal–External control belief scale (IE-4^28^, “Whether in my private life or at work, my life is largely determined by others.” on a scale ranging from 1 “does not apply at all” to 5 “applies completely”. Need for Cognition is measured with one item from the NFC-K^29^ “I like my life to be filled with difficult tasks to solve” on a scale ranging from 1 “completely disagree” to 7 “completely agree”. Trust in Intuition was measured with one Item from the Rational-Experiential Inventory^30^ “I trust my own intuition” on a scale from 1 “does not apply at all” to 5 “applies completely”. Optimism was measured with one item from the Optimism–Pessimism scale^31^, “Optimists are people who look to the future with confidence and mostly expect good things. Please rate yourself: How optimistic are you in general?” on a scale ranging from 1 “not at all optimistic” to 7 “very optimistic”. Psychological resilience was measured with a German version of the Brief Resilience Scale (BRS)^32^.

### Analyses

We first tested whether our samples differ significantly regarding age, gender, education or vaccination status to ensure comparability. To provide an overview of descriptive measures as well as exploratory group differences between vaccinated and unvaccinated participants in our sample regarding our core variables, we performed *t*-Tests. As preregistered (https://doi.org/10.17605/OSF.IO/HWVK9), we tested our hypotheses via multiple logistic regression analyses using vaccination as social norm, control beliefs, fear of COVID-19 and fear of vaccinations to predict vaccination status. We dummy-coded vaccination status (vaccinated = 0; unvaccinated = 1), meaning odds ratios significantly larger than 1 indicate an increased likelihood of being unvaccinated. To ensure the robustness of these results, we repeated the analyses controlling for wellbeing, need for cognition, trust in own intuition, optimism, age, and gender as possible covariates. We then perform moderated logistic regression analyses using the same set of variables and adding the interaction terms fear of COVID-19 × resilience as well as fear of a COVID-19 vaccination x resilience to predict vaccination status. We first mean-centered the relevant predictor variables (fear of COVID-19, fear of a COVID-19 vaccination, resilience) and added the interaction terms fear of COVID-19 × resilience as well as fear of a COVID-19 vaccination x resilience to the regression model without control variables. Analyses were performed in SPSS Statistics version 26.

## Supplementary Information

Supplementary Tables.

## Data Availability

The datasets used and/or analysed during the current study available from the corresponding author on reasonable request.
