# Supplementary material for: Fear of COVID-19 disease and vaccination as predictors of vaccination status
Source: Sci Rep. 2023 May 31;13:8865. doi: 10.1038/s41598-023-35064-0 (PMC10231848; doi:10.1038/s41598-023-35064-0)
Supplement: Supplementary file 1 — Supplementary Tables. [file 41598_2023_35064_MOESM1_ESM.docx]

# Supplementary Information

| **Supplementary Table 1 Logistic regression analysis to predict vaccination status including covariates** | | | | | | | |
| --- | --- | --- | --- | --- | --- | --- | --- |
|  |  |  |  |  |  | **95% CI** | |
| **Variable** | ***B*** | ***SE*** | **Wald** | ***p*** | ***OR*** | **Lower bound** | **Upper bound** |
| **Sample 1** |  |  |  |  |  |  |  |
| Fear of COVID-19 | -0.29 | 0.07 | 18.40 | <.001 | 0.750 | 0.658 | 0.855 |
| Fear of COVID-19 vaccination | 0.53 | 0.06 | 81.78 | <.001 | 1.691 | 1.509 | 1.895 |
| Vaccination as social norm | -0.35 | 0.07 | 25.40 | <.001 | 0.704 | 0.615 | 0.807 |
| Control belief | -0.16 | 0.12 | 1.97 | .848 | 0.848 | 0.674 | 1.067 |
| Wellbeing | 0.00 | 0.00 | 1.15 | .283 | 0.995 | 0.987 | 1.004 |
| Trust in own intuition | 0.49 | 0.16 | 9.52 | .002 | 1.628 | 1.195 | 2.218 |
| Need for cognition | 0.01 | 0.08 | 0.01 | .913 | 1.009 | 0.863 | 1.179 |
| Optimism | -0.05 | 0.09 | 0.28 | .595 | 0.953 | 0.797 | 1.139 |
| Age | -0.01 | 0.01 | 0.52 | .472 | 0.994 | 0.978 | 1.010 |
| Gender | 0.00 | 0.25 | 0.00 | .988 | 0.996 | 0.607 | 1.635 |
| Nagelkerke R^2^ | .448 |  |  |  |  |  |  |
|  |  |  |  |  |  |  |  |
| **Sample 2** |  |  |  |  |  |  |  |
| Fear of COVID-19 | -0.23 | 0.08 | 8.64 | .003 | 0.795 | 0.682 | 0.926 |
| Fear of COVID-19 vaccination | 0.65 | 0.06 | 103.50 | <.001 | 1.922 | 1.695 | 2.180 |
| Vaccination as social norm | -0.34 | 0.08 | 20.44 | <.001 | 0.711 | 0.613 | 0.824 |
| Control belief | -0.10 | 0.13 | 0.62 | .431 | 0.905 | 0.705 | 1.161 |
| Wellbeing | 0.00 | 0.01 | 0.00 | .997 | 1.000 | 0.990 | 1.010 |
| Trust in own intuition | 0.00 | 0.15 | 0.00 | .993 | 1.001 | 0.747 | 1.342 |
| Need for cognition | -0.02 | 0.09 | 0.04 | .839 | 0.982 | 0.820 | 1.174 |
| Optimism | 0.08 | 0.10 | 0.55 | .457 | 1.080 | 0.882 | 1.321 |
| Age | 0.01 | 0.01 | 0.31 | .578 | 1.005 | 0.987 | 1.023 |
| Gender | -0.14 | 0.27 | 0.27 | .603 | 0.868 | 0.509 | 1.480 |
| Nagelkerke R^2^ | .471 |  |  |  |  |  |  |
|  |  |  |  |  |  |  |  |
| **Sample 3** |  |  |  |  |  |  |  |
| Fear of COVID-19 | -0.31 | 0.09 | 13.24 | <.001 | 0.732 | 0.619 | 0.866 |
| Fear of COVID-19 vaccination | 0.62 | 0.06 | 91.55 | <.001 | 1.861 | 1.639 | 2.113 |
| Vaccination as social norm | -0.36 | 0.08 | 22.53 | <.001 | 0.698 | 0.602 | 0.810 |
| Control belief | 0.13 | 0.14 | 0.83 | .363 | 1.136 | 0.863 | 1.497 |
| Wellbeing | -0.01 | 0.01 | 1.86 | .172 | 0.992 | 0.981 | 1.003 |
| Trust in own intuition | 0.40 | 0.18 | 4.79 | .029 | 1.492 | 1.043 | 2.135 |
| Need for cognition | -0.09 | 0.10 | 0.71 | .400 | 0.917 | 0.749 | 1.122 |
| Optimism | -0.16 | 0.12 | 1.81 | .179 | 0.849 | 0.670 | 1.077 |
| Age | 0.00 | 0.01 | 0.15 | .703 | 1.004 | 0.985 | 1.023 |
| Gender | 0.38 | 0.29 | 1.66 | .198 | 1.461 | 0.820 | 2.604 |
| Nagelkerke R^2^ | .522 |  |  |  |  |  |  |
| Note. *B* = Regression weights. *SE* = Standard error of regression weights. *OR* = odds ratios. | | | | | | | |

| **Supplementary Table 2** |  |  |  |  |  |  |  |
| --- | --- | --- | --- | --- | --- | --- | --- |
|  |  |  |  |  |  | **95% CI** | |
| **Variable** | ***B*** | ***SE*** | **Wald** | ***p*** | ***OR*** | **Lower bound** | **Upper bound** |
| **Sample 1** |  |  |  |  |  |  |  |
| Fear of COVID-19 | -0.32 | 0.07 | 20.35 | <.001 | 0.730 | 0.636 | 0.837 |
| Fear of COVID-19 vaccination | 0.55 | 0.06 | 82.84 | <.001 | 1.727 | 1.535 | 1.942 |
| Vaccination as social norm | -0.34 | 0.07 | 23.89 | <.001 | 0.710 | 0.619 | 0.815 |
| Control belief | -0.18 | 0.12 | 2.34 | .126 | 0.835 | 0.663 | 1.052 |
| Wellbeing | -0.01 | 0.01 | 1.26 | .262 | 0.994 | 0.985 | 1.004 |
| Trust in own intuition | 0.47 | 0.16 | 8.95 | .003 | 1.602 | 1.176 | 2.182 |
| Need for cognition | -0.01 | 0.08 | 0.01 | .913 | 0.991 | 0.846 | 1.162 |
| Optimism | -0.06 | 0.09 | 0.40 | .528 | 0.943 | 0.787 | 1.131 |
| Age | -0.01 | 0.01 | 0.47 | .492 | 0.994 | 0.978 | 1.011 |
| Gender | 0.02 | 0.25 | 0.01 | .935 | 1.021 | 0.620 | 1.681 |
| Resilience | -0.18 | 0.23 | 0.64 | .423 | 0.834 | 0.535 | 1.300 |
| Fear of COVID-19 x Resilience | -0.25 | 0.09 | 8.85 | .003 | 0.775 | 0.655 | 0.917 |
| Fear of COVID-19 vaccination x Resilience | -0.02 | 0.07 | 0.11 | .738 | 0.977 | 0.853 | 1.119 |
| Nagelkerke R^2^ | .461 |  |  |  |  |  |  |
|  |  |  |  |  |  |  |  |
| **Sample 2** |  |  |  |  |  |  |  |
| Fear of COVID-19 | -0.22 | 0.08 | 7.37 | .007 | 0.806 | 0.689 | 0.942 |
| Fear of a COVID-19 vaccination | 0.66 | 0.07 | 99.35 | <.001 | 1.933 | 1.698 | 2.201 |
| Vaccination as social norm | -0.35 | 0.08 | 20.64 | <.001 | 0.702 | 0.602 | 0.818 |
| Control belief | -0.10 | 0.13 | 0.53 | .468 | 0.909 | 0.703 | 1.176 |
| Wellbeing | 0.00 | 0.01 | 0.01 | .941 | 1.000 | 0.990 | 1.011 |
| Trust in own intuition | 0.00 | 0.15 | 0.00 | .993 | 0.999 | 0.740 | 1.348 |
| Need for cognition | -0.01 | 0.09 | 0.01 | .907 | 0.989 | 0.823 | 1.188 |
| Optimism | 0.06 | 0.11 | 0.34 | .560 | 1.064 | 0.863 | 1.312 |
| Age | 0.00 | 0.01 | 0.22 | .640 | 1.004 | 0.986 | 1.023 |
| Gender | -0.09 | 0.28 | 0.11 | .742 | 0.914 | 0.533 | 1.567 |
| Resilience | 0.34 | 0.29 | 1.41 | .234 | 1.405 | 0.802 | 2.460 |
| Fear of COVID-19 x Resilience | 0.09 | 0.09 | 1.02 | .311 | 1.097 | 0.917 | 1.312 |
| Fear of COVID-19 vacciation x Resilience | -0.09 | 0.07 | 1.47 | .225 | 0.916 | 0.795 | 1.056 |
| Nagelkerke R^2^ | .476 |  |  |  |  |  |  |
|  |  |  |  |  |  |  |  |
| **Sample 3** |  |  |  |  |  |  |  |
| Fear of COVID-19 | -0.30 | 0.09 | 11.935 | .001 | 0.740 | 0.623 | 0.878 |
| Fear of COVID-19 vaccination | 0.63 | 0.07 | 89.024 | <.001 | 1.879 | 1.648 | 2.142 |
| Vaccination as social norm | -0.35 | 0.08 | 20.213 | <.001 | 0.708 | 0.609 | 0.823 |
| Control belief | 0.15 | 0.14 | 1.124 | .289 | 1.166 | 0.878 | 1.549 |
| Wellbeing | -0.01 | 0.01 | 0.982 | .322 | 0.994 | 0.982 | 1.006 |
| Trust in own intuition | 0.38 | 0.19 | 4.113 | .043 | 1.458 | 1.013 | 2.098 |
| Need for cognition | -0.11 | 0.10 | 1.026 | .311 | 0.899 | 0.732 | 1.104 |
| Optimism | -0.17 | 0.12 | 1.896 | .169 | 0.844 | 0.662 | 1.075 |
| Age | 0.00 | 0.01 | 0.110 | .741 | 1.003 | 0.984 | 1.023 |
| Gender | 0.39 | 0.30 | 1.758 | .185 | 1.482 | 0.828 | 2.653 |
| Resilience | 0.25 | 0.30 | 0.689 | .407 | 1.278 | 0.716 | 2.282 |
| Fear of COVID-19 x Resilience | 0.08 | 0.10 | 0.620 | .431 | 1.084 | 0.887 | 1.326 |
| Fear of COVID-19 vacciation x Resilience | 0.02 | 0.08 | 0.038 | .845 | 1.016 | 0.868 | 1.188 |
| Nagelkerke R^2^ |  |  |  |  |  |  |  |
| Note. *B* = Regression weights. *SE* = Standard error of regression weights. *OR* = odds ratios. | | | | | | | |
